# *HANABA TARANU* regulates the shoot apical meristem and leaf development in cucumber (*Cucumis sativus* L.)

**DOI:** 10.1093/jxb/erv409

**Published:** 2015-08-28

**Authors:** Lian Ding, Shuangshuang Yan, Li Jiang, Meiling Liu, Juan Zhang, Jianyu Zhao, Wensheng Zhao, Ying-yan Han, Qian Wang, Xiaolan Zhang

**Affiliations:** ^1^Department of Vegetable Sciences, Beijing Key Laboratory of Growth and Developmental Regulation for Protected Vegetable Crops, China Agricultural University, Beijing 100193, China; ^2^Department of Plant Science and Technology, Beijing University of Agriculture, Beijing, 102206, China

**Keywords:** *CsHAN*, *CsSTM*, *CsWUS*, cucumber, leaf development, shoot apical meristem.

## Abstract

The shoot apical meristem (SAM) is essential for continuous organogenesis in higher plants, while the leaf is the primary source organ and the leaf shape directly affects the efficiency of photosynthesis. *HANABA TARANU* (*HAN*) encodes a GATA3-type transcription factor that functions in floral organ development, SAM organization, and embryo development in *Arabidopsis*, but is involved in suppressing bract outgrowth and promoting branching in grass species. Here the function of the *HAN* homologue *CsHAN1* was characterized in cucumber, an important vegetable with great agricultural and economic value. *CsHAN1* is predominantly expressed at the junction of the SAM and the stem, and can partially rescue the *han-2* floral organ phenotype in *Arabidopsis*. Overexpression and RNAi of *CsHAN1* transgenic cucumber resulted in retarded growth early after embryogenesis and produced highly lobed leaves. Further, it was found that *CsHAN1* may regulate SAM development through regulating the *WUSCHEL* (*WUS*) and *SHOOT MERISTEMLESS* (*STM*) pathways, and mediate leaf development through a complicated gene regulatory network in cucumber.

## Highlight

Functional analysis of cucumber *CsHAN1* showed that it regulates meristem development through *WUSCHEL* and *SHOOT MERISTEMLESS* pathways, and mediates leaf development through a complicated gene regulatory network in cucumber.

## Introduction

The shoot apical meristem (SAM) is crucial for continuous organogenesis in higher plants. All the aerial organs including leaves, flowers, and stems are initiated from the SAM. The SAM is generally established during embryogenesis with a dome-shaped morphology, and can be divided into three functional zones: (i) the central zone with self-maintaining stem cells at the centre of the SAM; (ii) the peripheral zone where the lateral organ primordia are initiated from the shoulder of the SAM; and (iii) the rib zone in which stem tissue is specified beneath the central zone of the SAM (Steeves and Sussex, 1989; Fletcher, 2002; Tucker and Laux, 2007). Two independent pathways have been identified to be required for meristem establishment and maintenance in *Arabidopsis*, one is the *WUSCHEL* (*WUS*)–*CLAVATA* (*CLV*) pathway (Brand *et al.*, 2000; Schoof *et al.*, 2000). WUS, a homeodomain transcription factor, is expressed in the centre of the SAM, called the organizing centre, and functions to promote meristematic cell fate (Mayer *et al.*, 1998). Mutation in *WUS* leads to a premature SAM with no ability to self-maintain the stem cells (Laux *et al.*, 1996). CLV3, a signalling peptide, directly binds to the plasma membrane-localized receptor-like kinases CLV1 or CLV2/CRN complex and transmits a signal that restricts *WUS* expression, while *WUS* promotes the expression of *CLV3* in the stem cells as a feedback loop (Fiers *et al.*, 2005; Ito *et al.*, 2006; Kondo *et al.*, 2006; Ogawa *et al.*, 2008; Bleckmann *et al.*, 2010; Yadav *et al.*, 2011). *SHOOT MERISTEMLESS* (*STM*) is the other pathway that is essential for meristem maintenance. *STM*, a *KNOTTED1-LIKE HOMEOBOX* (*KNOX*) gene, is expressed throughout the SAM but is excluded from the organ primordia that function to maintain the undifferentiated cells in the SAM (Endrizzi *et al.*, 1996; Long *et al.*, 1996; Lenhard *et al.*, 2002). KNAT1/BREVIPEDICELLUS (BP), another member of the KNOX family, plays a role in meristem maintenance partially redundant with STM (Byrne *et al.*, 2002; Douglas *et al.*, 2002).

Leaf is the primary source organ, and the leaf shape directly affects the efficiency of photosynthesis (Tsukaya, 2006; Nicotra *et al.*, 2008). The leaf primordium is initiated from the peripheral zone of the SAM, in which *STM* is down-regulated (Long *et al.*, 1996) and *ASYMMETRIC LEAVES 1* and *2* (*AS1/2*) are up-regulated (Ori *et al.*, 2000; Guo *et al.*, 2008). Leaves of *as1* and *as2* mutants are downward curling with asymmetric lobes and short petioles (Byrne *et al.*, 2000; Iwakawa *et al.*, 2002; Iwakawa *et al.*, 2007). AS1 and AS2 form a protein complex that directly represses *BP* and *KNAT2* transcription (Guo *et al.*, 2008). Consistently, ectopic expression of *KNOX* genes results in lobed leaves in simple leaf species, and super-compoundness in compound leaf species such as tomato (Lincoln *et al.*, 1994; Chuck *et al.*, 1996; Janssen *et al.*, 1998; Hake *et al.*, 2004; Belles-Boix *et al.*, 2006). Further functional studies assessed a key role for *KNOX* genes in leaf shape determination (Hay and Tsiantis, 2010; Di Giacomo *et al.*, 2013; Bar and Ori, 2015). Several additional regulators have been found to mediate leaf shape development. For example, mutation in SERRATE (SE), a zinc finger protein involving in a miRNA gene silencing pathway, results in serrated leaves in *Arabidopsis* (Prigge and Wagner, 2001). *Cap Binding Protein 20* (*CBP20*) encodes the 20kDa subunit of the nuclear mRNA cap-binding complex (nCBC), and a *cpb20* mutant shows a serrated leaf margin (Papp *et al.*, 2004). Mutation of *ARGONAUTE1* (*AGO1*), a key player in transgene-induced post-transcriptional gene silencing, also leads to serrated leaves (Bohmert *et al.*, 1998; Morel *et al.*, 2002). *AGO10*/*PINHEAD* (*PNH*), another AGO protein gene, represses the accumulation of miR165/166, thereby affecting the establishment of leaf polarity (Liu *et al.*, 2009). *JAGGED* (*JAG*), a C2H2-type zinc finger transcription factor gene, is expressed in the initiating lateral organ primordia and is essential for proper leaf shape. *jag* mutants show narrow and serrated leaves, and the gain-of-function mutant *jag*-5D has bract-like organs subtending most flowers (Dinneny *et al.*, 2004; Ohno *et al.*, 2004). Boundary genes *CUP-SHAPED COTYLEDON* (*CUC1*, *2*, and *3*) were initially identified by defective SAM development and organ fusion (Aida *et al.*, 1999; Aida and Tasaka, 2006). Recently, *CUC* genes have been shown to play an important role in leaf margin development in both simple and compound leaf species and to act downstream of *KNOX* transcription factor genes (Nikovics *et al.*, 2006; Blein *et al.*, 2008; Berger *et al.*, 2009; Bilsborough *et al.*, 2011; Hasson *et al.*, 2011; Spinelli *et al.*, 2011). In *Arabidopsis*, genetic interactions among these different regulators lead to increased dissection of the *Arabidopsis* leaf margins (Blein *et al*., 2013).

*HANABA TARANU* (*HAN*) is a boundary gene that regulates SAM organization and flower organ development in *Arabidopsis* (Zhao *et al.*, 2004). *HAN* encodes a GATA3 transcription factor that is expressed in the boundaries between the meristem and developing organ primordia, the boundaries between different floral whorls, as well as the junctional domain between the SAM and the stem (Zhao *et al.*, 2004). Mutation of *HAN* leads to fused sepals, reduced floral organs, and a flatter SAM (Zhao *et al.*, 2004). Previous studies showed that *HAN* and three GATA3 family genes, *HANL2* (*HAN-LIKE 2*), *GNC* (*GATA, NITRATE-INDUCIBLE*, *CARBON-METABOLISM-INVOLVED*), and *GNL* (*GNC-LIKE*), form a negative feedback loop to regulate flower development (Zhang *et al.*, 2013). The functions of HAN homologues are divergent in different species. For example, *HAN* homologues such as *TASSEL SHEATH1* (*TSH1*) in maize (*Zea mays*), *NECK LEAF1* (*NL1*) in rice (*Oryza sativa*), and *THIRD OUTER GLUME* (*TRD*) in barley (*Hordeum vulgare*) are involved in repressing bract outgrowth and promoting branching (Wang *et al.*, 2009; Whipple *et al.*, 2010).

In this study, the function of *HAN* was explored in cucumber (*Cucumis sativus* L.), a globally cultivated vegetable that is of important economic and nutritional value (Huang *et al.*, 2009). Unlike the model plant *Arabidopsis* and most crops, cucumber is a typical unisexual plant with indeterminate growth, continuously producing leaves and male or female flowers simultaneously (Malepszy and Niemirowicz-Szczytt, 1991; Kater *et al.*, 2001; Hao *et al.*, 2003; Bai *et al.*, 2004). Two *HAN* homologous genes were identified in cucumber, and the function of *CsHAN1* was characterized in detail. *CsHAN1* is predominantly expressed at the junction of the SAM and the stem, and can partially rescue the *han-2* floral organ phenotype in *Arabidopsis*. Overexpression or down-regulation of *CsHAN1* in the transgenic cucumber plants led to retarded growth and lobed leaves. Further, it was found that *CsHAN1* may regulate SAM development through bridging the *WUS* and *STM* pathways, and mediate leaf margin development through a complicated gene regulatory network in cucumber.

## Materials and methods

### Plant materials and growth conditions

Cucumber (*Cucumis stativus* L.) inbred line R1407, which is a northern China type cucumber with dark green fruits similar to the sequenced line 9930, was used in this study. The cucumber seedlings were grown in a growth chamber under 16h/8h and 25 °C/18 °C day/night until the two true-leaf stage, and the cucumber plants were then transferred to a greenhouse in the experimental field of China Agricultural University in Beijing. Pest control and water management were carried out according to standard protocols. The *Arabidopsis thaliana* Landsberg *erecta* (L*er*) and Columbia (Col) ecotypes, and the mutant alleles *han-2(Ler*) were described previously (Zhao *et al.*, 2004; Zhang *et al.*, 2013) and obtained from the Meyerowitz lab stock collection. The mutant allele *han-2(Col*) was obtained by crossing *han-2(Ler*) to Col followed by six generations of selfing. The *Arabidopsis* plants were grown in a growth chamber under 16h light/8h dark at 22 °C.

### Gene cloning and phylogenetic analysis

Total RNA was extracted from the cucumber floral buds using a Quick RNA isolation Kit (Waryoung, China), and cDNA was synthesized using a Promega reverse transcriptase kit (Promega, USA). The coding sequences (CDS) of *CsHAN1* and *CsHAN2* were obtained using gene-specific primers (Supplementary Table S1 available at *JXB* online). The gene structure analysis was performed using online software GSDS 2.0 (http://gsds.cbi.pku.edu.cn/). The amino acid sequences of related HAN proteins in other species were obtained by BLAST searches (http://www.ncbi.nlm.nih.gov/BLAST/). Protein alignment of CsHAN and related HANs was performed using ClustalW in the MEGA5 software package, and the boxes were drawn using the BoxShade web site (http://www.ch.embnet.org/software/BOX_form.html). The phylogenetic analysis based on amino acid sequences was performed using the Neighbor–Joining (NJ) method with 1000 bootstrap replicates through MEGA5 software (Saitou and Nei, 1987).

### Quantitative real-time PCR

Total RNA was extracted from different cucumber tissues or *Arabidopsis* inflorescences using a Quick RNA isolation Kit (Waryoung, China), and cDNA was synthesized using a Promega reverse transcriptase kit (Promega, USA). An ABI PRISM 7500 Real-Time PCR System (Applied Biosystems, USA) was used for quantitative real-time reverse transcription–PCR (qRT–PCR) experiments. Three biological and three technical replicates (3×3) were performed for each gene. The cucumber *Ubiquitin extension protein* (*UBI-ep*) gene and the *Arabidopsis ACTIN2* gene were used as internal references to normalize the expression data. The standard deviation was calculated between three biological replicates, using the average of the three technical replicates for each biological sample. The gene-specific primers are listed in Supplementary Table S1 at *JXB* online.

### *In situ* hybridization

Cucumber shoot apexes of 6-, 12-, and 15-day-old seedlings, male and female buds, and young fruits from 0.8cm to 2.8cm were fixed in 3.7% formalin–acetic acid–alcohol (FAA), and *in situ* hybridization was performed as described previously (Zhang *et al.*, 2013). Sense and antisense probes were synthesized by PCR amplification using SP6 and T7 RNA polymerase, respectively. Probes of *CsWUS*, *CsSTM*, and *CsBP* were designed according to the specific gene fragments. The primers for probe generation are listed in Supplementary Table S1 at *JXB* online.

### Ectopic expression of *CsHAN1* in *Arabidopsis*

To make the *CsHAN1* overexpression construct, the full-length *CsHAN1* CDS were amplified and cloned into the binary vector pBI121 through *Xba*I and *Sma*I sites. The recombinant plasmids were introduced into *Agrobacterium* by electroporation and then transformed into wild-type (WT) and *han-2* mutant plants through the floral dip method (Clough and Bent, 1998). The transgenic plants were screened on Murashige and Skoog (MS) medium with 40mg l^–1^ kanamycin. The primers for vector construction are listed in Supplementary Table S1 at *JXB* online.

### Cucumber transformation

The same *CsHAN1* overexpression construct was used for cucumber transformation. To generate *CsHAN1-RNAi* transgenic plants, the 258bp sense and antisense fragments from the 3′ end of *CsHAN1* were amplified using gene-specific primers containing *Spe*I(5′ end)/*Sac*I(3′ end) and *Bam*HI(5′ end)/*Kpn*I(3′ end) sites, respectively. The two fragments were inserted into the RNAi-1 vector, and the empty RNAi-1 vector was used as a transformation control. The resultant *CsHAN1-RNAi* construct and empty RNAi-1 vector were then delivered into *Agrobacterium* by electroporation and transformed into the cucumber inbred line R1407 line using the cotyledon transformation method as previously described (Wang *et al.*, 2014). The primers containing the restriction enzyme cutting sites are listed in Supplementary Table S1 at *JXB* online.

### Paraffin sections

Young cucumber seeds at 16 d after fertilization were fixed, embedded, sectioned, and dewaxed as described (Jiang *et al.*, 2014). Sections of 8 μm thickness were mounted in neutral resins, and images were taken under a light microscope (D72, Olympus, Japan).

### Accession numbers

Sequence data in this paper can be found in the Cucumber Genome DataBase, TAIR, or GenBank under the following accession numbers: *CsHAN1* (Csa016191), *CsHAN2* (Csa012029), *CsPNH1* (Csa015921), *CsPNH2* (Csa004392), *CsAGO1* (Csa000946), *CsJAG* (Csa008074), *CsAS2* (Csa012250), *CsBP* (Csa009344), *CsKNAT2* (Csa013896), *CsKNAT6* (Csa011388), *CsWUS* (Csa000479), *CsSTM* (Csa000554), *AtHAN* (AT3G50870), *SE* (AT2G27100), *AGO1* (AT1G48410), *AS2* (AT1G65620), *KNAT2* (AT1G70510), *CPB20* (AT5G44200), *BP* (AT4G08150), *CUC3* (AT1G76420), *PNH* (AT5G43810), *JAG* (AT1G68480), *GNC* (AT5G56860), *GNL* (AT4G26150), *HvTRD* (GU722206), *OsNL1* (DQ784546), and *ZmTSH1* (AC199892.4_FG031).

## Results

### Isolation of the cucumber *CsHAN* genes

**Fig. 1.**
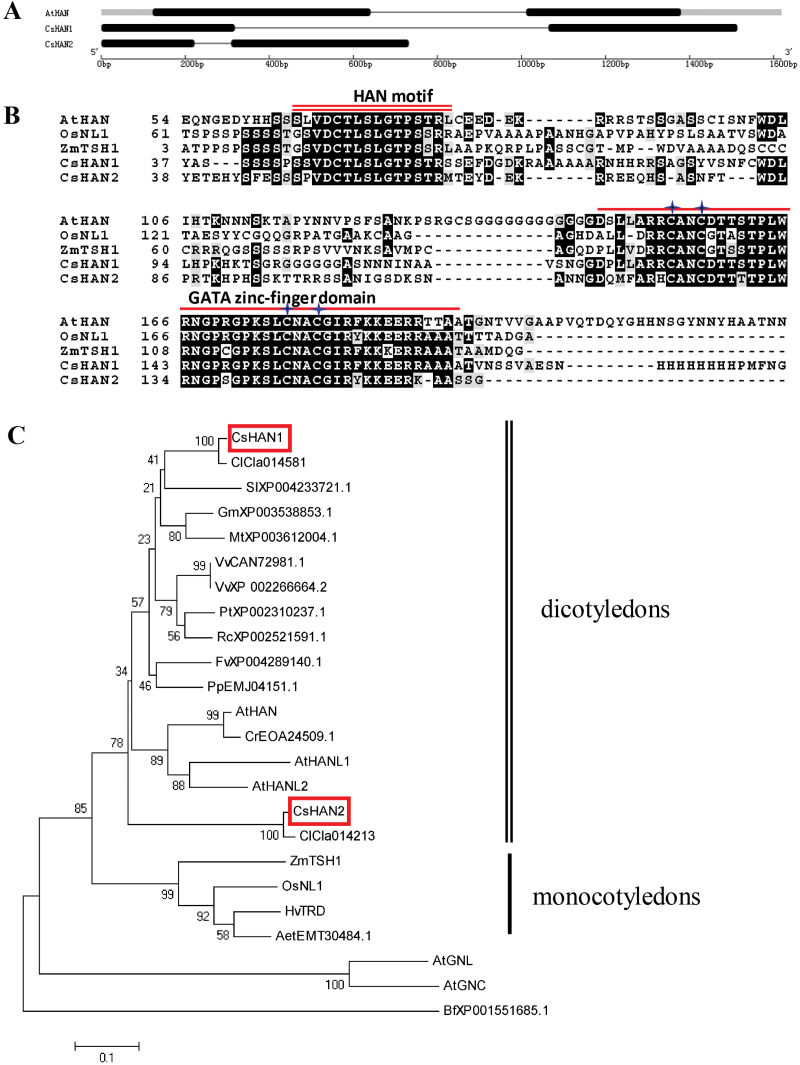
Gene structure and phylogenetic analyses of *CsHAN*. (A) Structural analysis of *HAN* genes in cucumber and *Arabidopsis*. Grey boxes represent the 3′- or 5′-untranslated regions, black boxes indicate the exon, and black lines represent the introns. *Cs*, *Cucumis sativus*; *At*, *Arabidopsis thaliana*. (B) Protein alignment of HANs from *Arabidopsis*, rice (*Oryza sativa*), maize (*Zea mays*), and cucumber. The single and double underlines indicate the conserved GATA zinc finger domain and HAN motif, respectively. The asterisks indicate the conserved cysteine residues present in type IV zinc finger domains (C-X2-C-X17-20-C-X2-C). (C) Phylogenetic analysis of *CsHAN* genes (boxed) and *HAN*-like genes. MEGA 5.0 software was used to construct the Neighbor–Joining tree. Homologues of *CsHAN* genes from 11 dicotyledon species (double underlines) and four monocotyledon species (single underline) were used for the analyses and formed distinct clades (dicotyledon group and monocotyledon group). Vv, *Vitis vinifera*; Cr, *Capsella rubella*; Pt, *Populus trichocarpa*; Rc, *Ricinus communis*; Fv, *Fragaria vesca* subsp. *vesca*; Gm, *Glycine max*; Pp, *Prunus persica*; Mt, *Medicago truncatula*; Sl, *Solanum lycopersicum*; Bf, *Botryotinia fuckeliana*; Aet, *Aegilops tauschii*; Cl, *Citrullus lanatus*; Hv, *Hordeum vulgare*, Zm, *Zea mays*; Os, *Oryza sativa*; At, *Arabidopsis thaliana*. (This figure is available in colour at *JXB* online.)

To identify the *HAN* homologues from cucumber, a BLAST search was performed in the Cucumber Genome DataBase (Huang *et al.*, 2009) based on the amino acid sequence information of *Arabidopsis* HAN. Two candidate genes, *Csa016191* and *Csa012029*, showed the highest similarity. A further BLAST search was performed in TAIR (http://www.arabidopsis.org/) using the two candidate gene, and both of them got the best hit to *Arabidopsis* HAN (AtHAN). Thus, *Csa016191* was named *CsHAN1* and *Csa012029* was named *CsHAN2*, respectively, and their CDS as well as their genomic sequence from the flower buds of cucumber line R1407 were cloned. Gene structure analysis showed that *CsHAN1* and *CsHAN2*, encoding 255 and 214 amino acids, respectively, contain two exons and one intron, consistent with the gene structure of *AtHAN* and *HAN* homologues (Zhao *et al.*, 2004; Wang *et al.*, 2009; Whipple *et al.*, 2010) (Fig. 1A). Previous studies showed that *HAN* encodes a GATA3-like transcription factor with a single zinc finger domain and a HAN motif (Whipple *et al.*, 2010). Protein alignment of HAN homologues from *Arabidopsis* (AtHAN), rice (OsNL1), maize (ZmTSH1), and cucumber (CsHAN1/2) was performed using ClustalW in the MEGA5 software. Despite CsHAN1 and CsHAN2 showing only 39.55% and 34.46% identity with AtHAN, respectively, the GATA zinc finger domain and the HAN motif are highly conserved (Fig. 1B).

Phylogenetic analysis of the deduced HAN proteins from various species was performed using the NJ method (Saitou and Nei, 1987). The phylogenetic tree showed that HAN homologues in the eudicot species form a distinct clade from those in the monocotyledon species such as rice, maize, and barley (Fig. 1C). In watermelon (*Citrullus lanatus*), another Cucurbitaceae species, there are also two HAN homologues (Cla014581and Cla014213) as well, and they formed two different clades with CsHAN1 and CsHAN2, respectively (Fig. 1C), implying that HAN homologues in Cucurbitaceae may have a distinct function from that in the model *Arabidopsis* plant. Given that CsHAN1 is more closely related to AtHAN than CsHAN2 (Fig. 1C), CsHAN1 was chosen and analysed in this study.

### Expression pattern of *CsHAN1* in cucumber

**Fig. 2.**
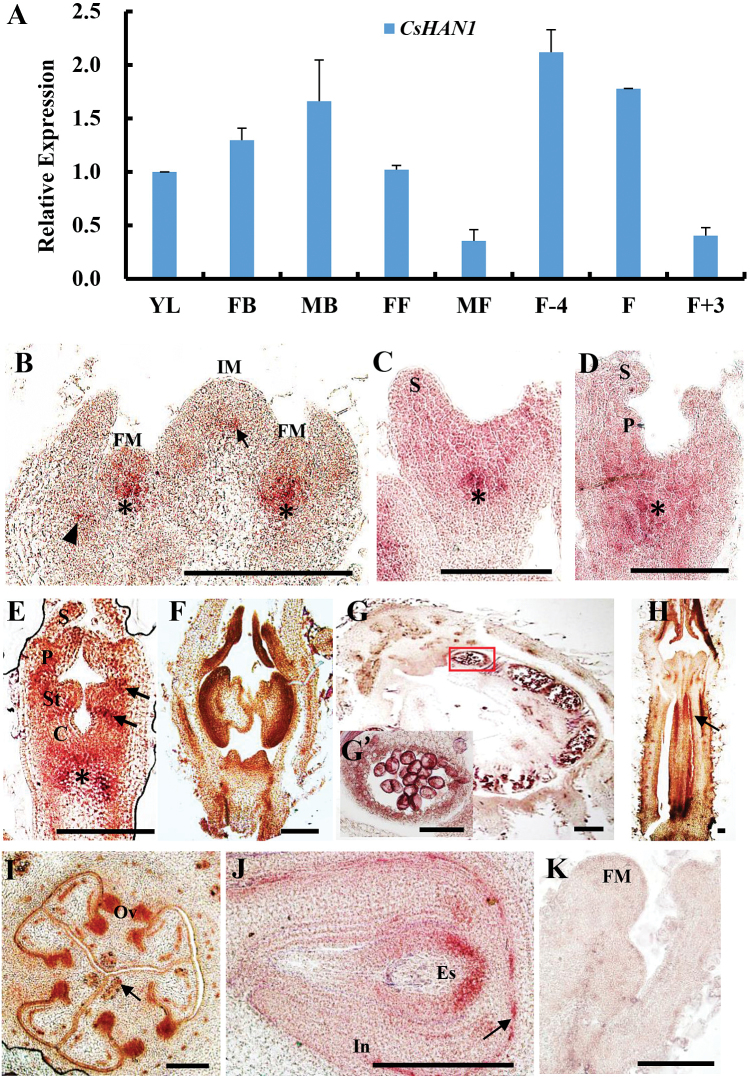
Expression analysis of *CsHAN1* in cucumber. (A) Quantitative RT–PCR (qRT–PCR) analysis of *CsHAN1* in different tissues of cucumber. YL, young leaves; FB, female buds; MB, male buds; FF, female flowers; MF, male flowers; F-4, young fruits 4 d before anthesis; F, fruit at anthesis; F+3, fruits 3 d after anthesis. The *Ubiquitin extension protein* (*UBI-ep*) gene was used as an internal reference to normalize the expression data. (B–K) *In situ* hybridization with the *CsHAN1* antisense probe (B–J) and sense probe (K). (B) In the ucumber shoot apex, *CsHAN1* is expressed in the junction region of the inflorescence meristem (IM) and stem (arrow), the junction regions of the floral meristem (FM) and stem (asterisk), and the axil of leaf primordia (arrowhead). (C–E) Floral buds at stage 2 (C), 3 (D), and 4 (E). Asterisks show the expression domain of *CsHAN1* at the junction of the meristem and stem, and arrows indicate the expression of *CsHAN1* at the boundary between the petal and stamen, and the boundary between the stamen and initiating carpel primordia. (F–G’) Male flowers at stage 9 (F) and stage 11 (G); (G’) is a high magnification view of the anther in (G). The signal of *CsHAN1* was detected in the developing anther, tapetum cell layer, and the uninuclear pollen. (H) Female flower in stage 8. *CsHAN1* is expressed in the ovary (arrow). (I, J) Cross-sections of the female ovary in stage 9 (I) and stage 10 (J) showing the expression domain of *CsHAN1* in the ovules and the base of the embryo sac. (K) No signal was found on hybridization with the sense *CsHAN1* probe. S, sepal; P, petal; St, stamen; C, carpel; Ov, ovule; In, integument; Es, embryo sac. Bar=100 μm. (This figure is available in colour at *JXB* online.)

The expression of *CsHAN1* was examined in different organs of cucumber through qRT–PCR (Fig. 2A). Total RNA was extracted from young leaves, female flower buds, male flower buds, female opening flowers, male opening flowers, and fruits at three different developmental stages. The data showed that *CsHAN1* has the highest level in the young fruits 4 d before anthesis, and exhibited the lowest level in the male opening flowers (Fig. 2A). The expression of *CsHAN1* in floral buds is higher than that in the opening flowers (Fig. 2A), suggesting that *CsHAN1* is more abundant in young tissues.

To investigate the spatial and temporal expression pattern of *CsHAN*, *in situ* hybridization was performed. The signal of *CsHAN1* is detected at the junction of the inflorescence meristem (IM) and the stem (arrow in Fig. 2B), the junction regions of the floral meristem (FM) and the stem (asterisks in Fig. 2C–E), and in the axil of leaf primordia (arrowhead in Fig. 2B). In addition, transcripts of *CsHAN1* are mainly concentrated in the boundary between petal primordia and stamen primordia, and the boundary between stamen primordia and carpal primordia in the stage 4 flower bud (arrows in Fig. 2E). In the male flower, *CsHAN1* is primarily expressed in the developing anthers at stage 9 (Fig. 2F), and then in the tapetum cell layer and uninuclear pollen at stage 11 (Fig. 2G). In the female flower, the expression domain of *CsHAN1* is mostly in the developing ovary (Fig. 2H), ovules (Fig. 2I), and the base of the embryo sac (Fig. 2J). *CsHAN1* is also strongly expressed in vascular tissues in all of the examined samples (arrows in Fig. 2I, J). No signal is detected upon hybridization with the sense *CsHAN1* probe (Fig. 2K).

### Ectopic expression of *CsHAN1* in *Arabidopsis*

**Fig. 3.**
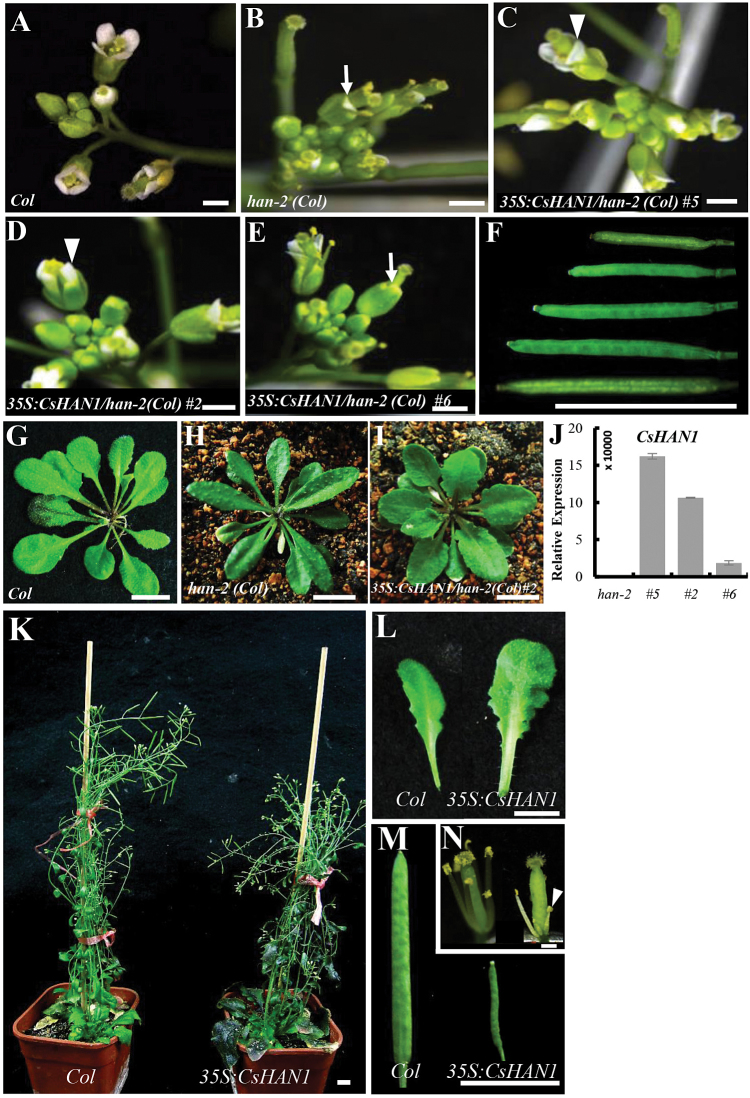
Ectopic expression of *CsHAN1* in *han-2* mutant and wild-type *Arabidopsis*. (A–E) The inflorescences of Col (A), *han-2(Col*) (B), *35S:CsHAN1/han-2(Col*) line 5 (C), *35S:CsHAN1/han-2(Col*) line 2 (D), and *35S:CsHAN1/han-2(Col*) line 6 (E). The arrows indicate the flowers with 1–2 petals, and the arrowheads show the flowers with 3–4 petals. (F) The siliques of WT, *35S:CsHAN1/han-2(Col*) line 5, *35S:CsHAN1/han-2(Col*) line 2, *35S:CsHAN1/han-2(Col*) line 6, and the *han-2* mutant at the same developmental stage (from bottom to top). (G–I) Rosette leaves of Col (G), *han-2(Col*) (H), and *35S:CsHAN1/han-2(Col*) line 2 (I) show the partially rescued leaf shape. (J) qRT–PCR analyses of *CsHAN1* in the three transgenic lines in the *han-2(Col*) background. *Arabidopsis ACTIN2* was used as an internal standard to normalize the templates. (K–N) The whole plants (K), rosette leaves (L), fruits (M), and flowers (N) of Col (left) and the *CsHAN1* overexpression line (right) in the Col background. Sepals and petals were removed in (N), and the white arrowhead shows the retarded stamen in the overexpression line. Bar=1mm. (This figure is available in colour at *JXB* online.)

To investigate the function of *CsHAN1*, *CsHAN1* was first ectopically introduced under the *Cauliflower mosaic virus* (CaMV) 35S promoter into the *han-2* mutant in the L*er* background. However, only two transgenic plants were produced, screened from ~3ml (1.1×10^4^) of seeds, and died without producing any seeds (Supplementary Fig. S1A, B at *JXB* online). This is similar to the overexpression of *AtHAN* itself in *Arabidopsis* (Zhao *et al.*, 2004). Next, *CsHAN1* was ectopically expressed in the *han-2* mutants in the Col background which displays similar reduced floral organs and decreased silique length to those in the L*er* background (Zhang *et al.*, 2013). Fortunately, 17 independent transgenic lines were produced, and the degree of rescue of the *han-2* mutant phenotype positively correlates with the ectopic *CsHAN1* expression (Fig. 3A–J) (investigation of the phenotype was performed in the T_2_ transgenic lines). For example, the number of petals rescued ranged from 1.9±1.1 in *han-2* to 3.6±0.5 in the strongest transgenic line 5, and 3.1±0.9 in the weakest line 6 (Fig. 3C–E; Table 1). Similarly, the length of the silique in the three *CsHAN1* transgenic lines was also increased, and line 5 recovered almost to the length of the WT (Fig. 3F). In addition, in contrast to the slightly serrated margin in Col, it was noticed that the rosette leaves of *han-2(Col*) are spindly with smooth margins and short petioles (Fig. 3G, H), and *CsHAN1* can rescue the smooth leaves to serrated upon ectopic expression in *Arabidopsis* (Fig. 3I). These results suggest that *CsHAN1* may play a role in regulation of flower organ and leaf shape development.

**Table 1.** CsHAN1 *can partly rescue the number of floral organs in the* han-2 *mutant in* Arabidopsis

Genotype	Sepal	Petal	Stamen	Carpal
Col	4.0±0.0	4.0±0.0	5.5±0.3	2.0±0.0
*han-2(Col*)	3.6±0.6	1.9±1.1	4.5±0.6	2.0±0.0
*35S:CsHAN1/han-2 #5*	3.8±0.5	3.6±0.5	4.5±0.5	2.0±0.0
*35S:CsHAN1/han-2 #2*	3.6±0.5	3.2±0.8	4.7±0.6	2.0±0.0
*35S:CsHAN1/han-2 #6*	3.5±0.8	3.1±0.9	4.4±0.5	2.0±0.0

The values shown are the means ±SE, *n*=30.

To explore further the function of *CsHAN1*, transgenic lines overexpressing *CsHAN1* in *Arabidopsis* WT Col were also generated. A total of 12 independent transgenic lines were obtained. Overexpression of *CsHAN1* leads to serrated leaves in both rosette leaves and cauline leaves, and produces short siliques that may partially result from the 28% short stamens that are not long enough to reach the stigma and/or immature anthers (Fig. 3K–N; Supplementary Fig. S1C at *JXB* online). However, the number of floral organs is unchanged in the *CsHAN1* overexpression lines.

### *CsHAN1* may be involved in shoot apical meristem development in cucumber

**Fig. 4.**
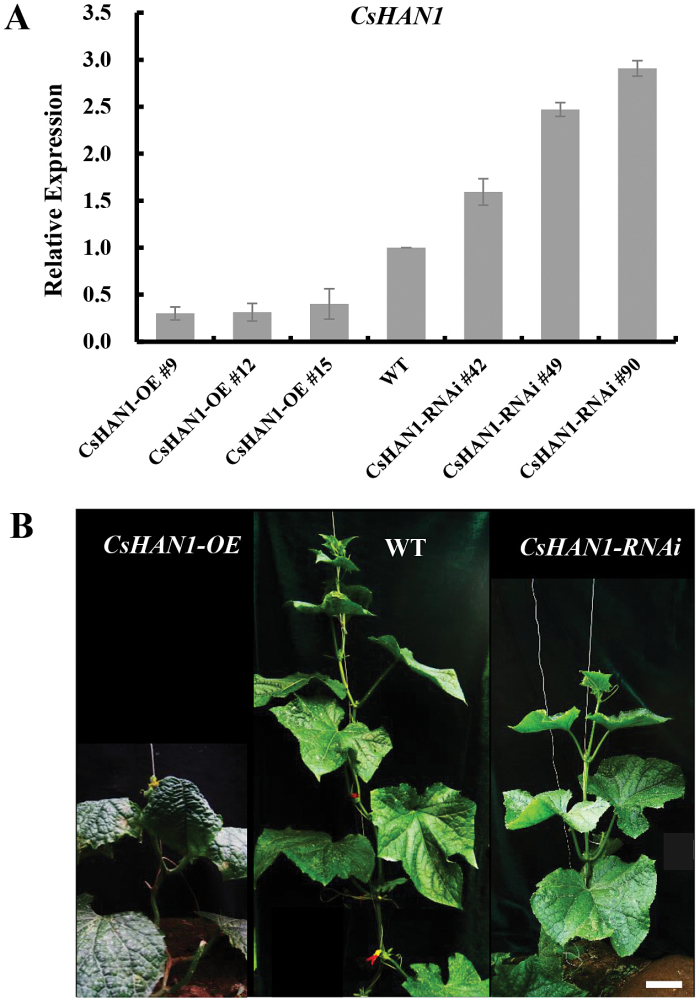
Phenotypes of transgenic cucumber. (A) qRT–PCR analyses of *CsHAN1* expression in transgenic overexpression and RNAi lines in cucumber. (B) Plant phenotypes of a transgenic *CsHAN1* overexpression line (left), the wild type (middle), and a *CsHAN1-RNAi* line (right) which are 50 days old. Bar=5cm. (This figure is available in colour at *JXB* online.)

**Fig. 5.**
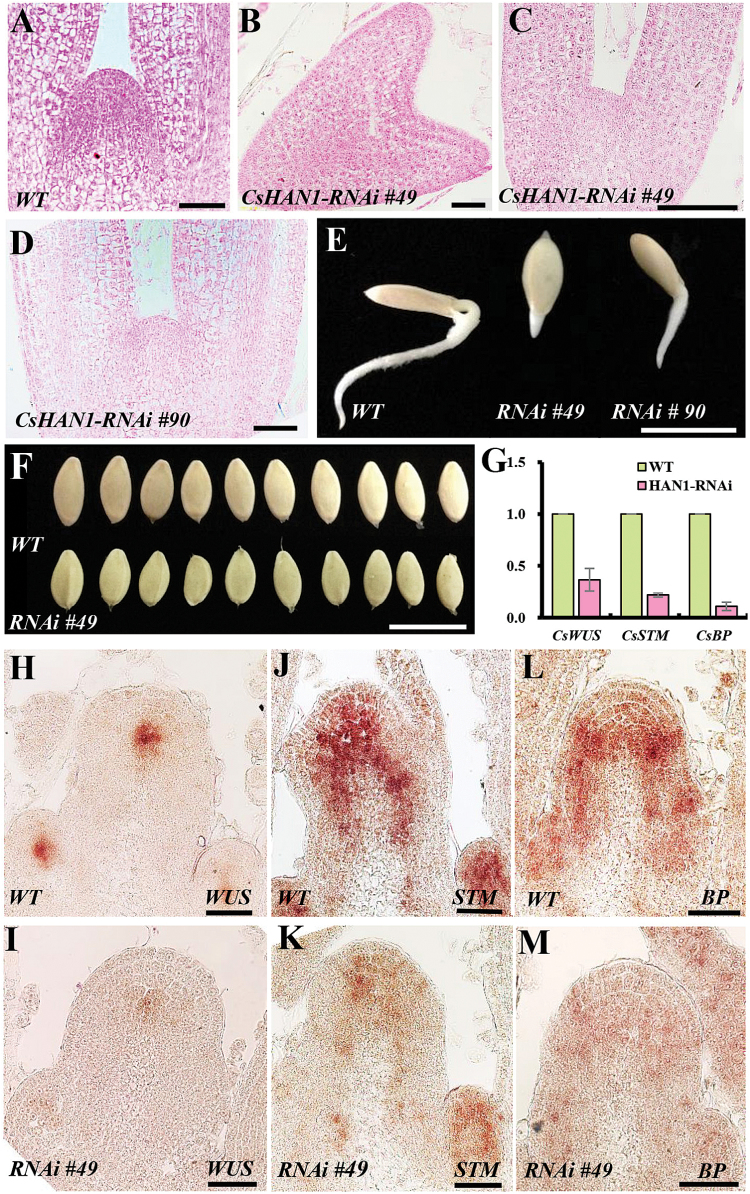
*CsHAN1* is required for shoot apical meristem development. (A–D) Embryo phenotypes in the normal torpedo stage of the wild type (A), the heart stage in the *CsHAN1-RNAi* line 49 (B), the retarded torpedo embryo in the *CsHAN1-RNAi* line 49 (C), and in the *CsHAN1-RNAi* line 90 (D) at 16 d after fertilization. Bar=50 μm. (E) Phenotypes of wild-type (left), *CsHAN1-RNAi* line 49 (middle), and *CsHAN1-RNAi* line 90 (right) seeds at 36h after germination. Bar=1cm. (F) Seed morphology in the WT and *CsHAN1-RNAi* line 49. Bar=1cm. (G) qRT–PCR analyses of *CsWUS*, *CsSTM*, and *CsBP* in the shoot apexes of the wild type and the *CsHAN1-RNAi* line. The *UBI-ep* gene was used as an internal reference to normalize the expression data. (H–M) The expression of *CsWUS* (H, I), *CsSTM* (J, K), and *CsBP* (L, M) in the wild type (H, J, L) and *CsHAN1-RNAi* line 49 (I, K, M) in the apex of 6-day-old seedlings as detected by *in situ* hybridization. Bar=50μm. (This figure is available in colour at *JXB* online.)

To understand further the function of *CsHAN1* in cucumber, the 35S promoter followed by the *CsHAN1* coding sequence (*CsHAN1-OE*) or the double-stranded RNAi construct containing the specific sequence of *CsHAN1* (*CsHAN1-RNAi*) was introduced into the cucumber inbred line R1407 through *Agrobacterium*-mediated cotyledon transformation, and positive transplants were selected based on antibiotic selection as well as PCR analyses using primers from the vector (Y. Zhang *et al.*, 2014; Cheng *et al.*, 2015). Nine *CsHAN1-OE* and 11 *CsHAN1-RNAi* independent T_0_ transgenic lines were obtained. Surprisingly, the expression of *CsHAN1* was down-regulated in the *CsHAN1-OE* lines whereas it was up-regulated in the *CsHAN1-RNAi* lines (Fig. 4A; Supplementary Fig. S2 at *JXB* online), which can be explained by co-suppression in the *CsHAN1-OE* lines and negative autoregulation of *HAN* in the *CsHAN1-RNAi* lines (Baudry *et al.*, 2006; Zhang *et al.*, 2013). Next three representative transgenic lines for each construct were selected for further characterization (Fig. 4A). In the three T_0_ *CsHAN1-OE* lines, transcripts of *CsHAN1* declined to 30, 31, and 40% in lines 9, 12, and 15, respectively, as compared with those in the empty vector (WT). In the T_0_ *CsHAN1-RNAi* lines, the expression of *CsHAN1* is up-regulated 1.6- to 3-fold (Fig. 4A). Line 9 of *CsHAN1-OE* grew slowly, with very few flower buds and lobed leaves (Fig. 4B; Supplementary Fig. S3), and it died after 3 months without generating any seeds. Despite *CsHAN1-OE* lines 12 and 15 producing several seeds, the resulting T_1_ plants display severely retarded growth and appear to be sterile (no seeds produced after pollination). Similarly, the T_0_ *CsHAN1-RNAi* line also grew slowly (Fig. 4B; Supplementary Fig. S3). The stunted transgenic lines suggest that *CsHAN1* may function in SAM development. To confirm this notion, embryo development was characterized in the T_2_ *CsHAN1-RNAi* lines (Fig. 5). In the WT, the cucumber embryo developed to the torpedo stage and the meristem protruded upward, forming a dome at 16 d after pollination (Fig. 5A) (Atsmon and Galun, 1960; Sun *et al.*, 2010). In the *CsHAN1-RNAi* line 49, ~30% of embryos remained at the heart stage (Fig. 5B), and 60% embryos were in the torpedo stage with a flat meristem (Fig. 5C). In the *CsHAN1-RNAi* line 90, although 75% of embryos were at the torpedo stage, the meristem was small or did not fully protrude (Fig. 5D). Next, the rate of seed germination was compared for 36h; the root of WT cucumbers was ~3cm long (Fig. 5E), while the root in the *CsHAN1-RNAi* lines just began to emerge or was <2cm long (Fig. 5E). The seed morphology was also affected in the *CsHAN1-RNAi* lines, with 39% of seeds obviously crapy and smaller than those in the WT (Fig. 5F). Therefore, *CsHAN1* can retard plant growth early after embryogenesis.

Given that *WUS* was shown to be a classical meristem marker that functions in specifying the stem cell identity in the shoot meristem, and *STM* and *BP* are KNOX family genes that promote meristem maintenance (Long *et al.*, 1996; Byrne *et al.*, 2002; Douglas *et al.*, 2002; Lenhard *et al.*, 2002), it was next explored whether *HAN* suppresses SAM development through these genes. qRT–PCR analyses showed that the expression of *CsWUS*, *CsSTM*, and *CsBP* was greatly decreased in the shoot apexes of *CsHAN1-RNAi* lines (Fig. 5G). *In situ* hybridization of *CsWUS*, *CsSTM*, and *CsBP* was also performed in the shoot apexes of *CsHAN1-RNAi* lines. The *CsWUS* signal was detected in the organizing centre of the SAM, IM, and FM in the WT, consistent with findings in other species (Fig. 5H), while the expression of *CsWUS* was significantly reduced in the *CsHAN1-RNAi* line (Fig. 5I). CsSTM is expressed throughout the SAM and FM but not in the organ primordia in the WT (Fig. 5J). In the *CsHAN1-RNAi* line, the *CsSTM* signal is also greatly decreased (Fig. 5K). Similarly, *CsBP* mRNA is detected at the base of the SAM and the cortex of the stem in the WT (Fig. 5L), and the transcript level of *CsBP* is greatly reduced in the *CsHAN1-RNAi* line (Fig. 5M). These data suggested that *CsHAN1* may regulate meristem development by mediating the expression of *CsWUS*, *CsSTM*, and *CsBP* in cucumber.

### *CsHAN1* regulates leaf shape development in cucumber

**Fig. 6.**
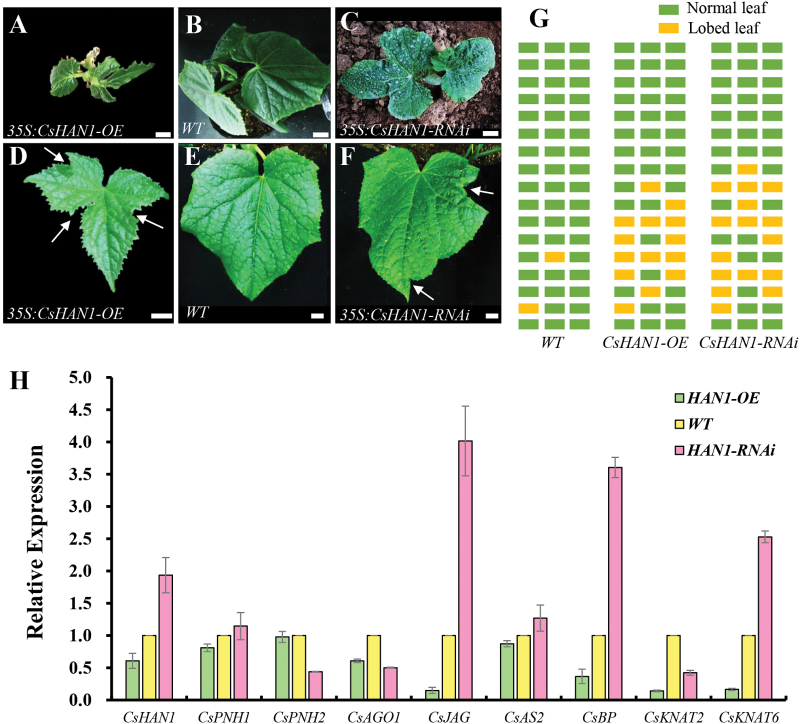
*CsHAN1* regulates leaf shape development. (A–F) Transgenic cucumber plants (A–C) and representative leaves (D–F) of a *CsHAN1* overexpression line (A, D), the wild type (B, E), and a *CsHAN1-RNAi* line (C, F) which are 20 days old. Arrows showed the notches. Bar=1cm. (G) Diagrammatic data show the position of lobed leaves in the WT and *CsHAN1* transgenic lines. Each column represents an individual plant, and each rectangle represents a node. (H) qRT–PCR analyses of leaf developmental genes in *CsHAN1* overexpression and RNAi lines. The cucumber *UBI-ep* gene was used as an internal reference to normalize the expression data, and the experiments were repeated in triplicate independent samples. Error bars represent the SE. (This figure is available in colour at *JXB* online.)

In addition to the retarded growth, another obvious phenotype in the *CsHAN1* transgenic cucumber was the lobed leaves (Fig. 6). In contrast to the palmate entire leaves in the WT, a high proportion of the leaves in both *CsHAN1*-*OE* and *CsHAN1-RNAi* lines were highly lobed (Fig. 6A–F), especially in leaves at the first 10 nodes, probably due to different penetrance and developmental cues at different nodes (Fig. 6G) (Weigel *et al.*, 1992; Ji *et al.*, 2011). To explore the mechanism by which *CsHAN1* regulates leaf shape development in cucumber, the known leaf developmental genes in cucumber were first isolated using a BLAST search, and then the expression in the fourth young leaves was examined by qRT–PCR in the T_2_ plants. The expression of *CsJAG*, *CsBP*, and *CsKNAT6* was down-regulated in the *CsHAN1*-*OE* lines and up-regulated in the *CsHAN1-RNAi* lines, whereas the expression of *CsAGO1* and *CsKNAT2* was reduced in both the *CsHAN1-OE* lines and *CsHAN1-RNAi* lines (Fig. 6H). The expression of *CsPNH2* was reduced >2-fold in the *CsHAN1-RNAi* lines, but was unchanged in the *CsHAN1*-*OE* lines (Fig. 6H). The expression of *CsPNH1* and *CsAS2* appears to be unaffected in both transgenic lines (Fig. 6H), suggesting that *CsHAN1* regulates leaf shape development through a complicated gene regulatory network in cucumber.

## Discussion

### *CsHAN1* may regulate shoot meristem development through regulating *WUS* and *STM* pathways in cucumber

In *Arabidopsis*, *WUS* and *STM* function in independent pathways and play essential roles for SAM establishment and maintenance (Lenhard *et al.*, 2002). Here it was found that both *CsHAN1*-*OE* and *CsHAN1-RNAi* lines displayed retarded growth, but *CsHAN1*-*OE* lines displayed a more severe phenotype than the *CsHAN1-RNAi* lines, probably due to the huge reduction caused by co-suppression in the *CsHAN1*-*OE* lines (Figs 4–6). *In situ* hybridization showed that the expression of *CsWUS* was significantly reduced in the *CsHAN1-RNAi* lines, despite the fact that the expression domain remained unchanged (Fig. 5H, I). However, the expression of *AtWUS* was diffused and shifted to the L2 or L1 layer in the *han-1* mutant plants in *Arabidopsis* (Zhao *et al.*, 2004), implying that *CsHAN1* and *AtHAN* may regulate *WUS* in a different way. In addition, embryo development in the *han-1* mutant was uncoordinated in *Arabidopsis*, resulting in misshapen embryos (Zhao *et al.*, 2004; Nawy *et al.*, 2010), whereas the embryo development in the *CsHAN1-RNAi* line was delayed, with no obvious change of embryo shape. There are two possibilities to explain this difference: one is that *CsHAN1* and *AtHAN* regulate embryo development through a distinct mechanism, and the other possibility is that the embryo defects in the *CsHAN1-RNAi* line were covered by *CsHAN1* autoregulation; the 1.6- to 3-fold increase of *CsHAN1* expression in the *CsHAN1-RNAi* line was within the buffer threshold that fails to produce any morphological defects in the embryo. A clean loss of function of *CsHAN1* like that in the *han-1* null allele would better elucidate the function of *CsHAN1* in embryo development in cucumber.

Further, it was found that the expression of *CsSTM* and *CsBP* was greatly reduced in the *CsHAN1-RNAi* lines (Fig. 5J–M). *STM* and *BP* both belong to the class 1 *KNOX* genes, and were shown to function redundantly in meristem maintenance in *Arabidopsis* (Byrne *et al.*, 2002; Douglas *et al.*, 2002; Lenhard *et al.*, 2002). Given that the expression domain of *CsHAN1* overlaps with that of *CsWUS*, *CsSTM*, and *CsBP* (Figs 2, 5), *CsHAN1* may regulate meristem development through physical interactions with *CsWUS* and *CsSTM*, bridging the previously speculated two parallel pathways in cucumber. Further studies using inducible *CsHAN1* lines and ChIP assay will be helpful to test the above hypothesis.

### Elaborate expression of *CsHAN1* is required for normal leaf shape development

Previous studies of HAN emphasized its role in flower and embryo development (Zhao *et al.*, 2004; Nawy *et al.*, 2010), while the function of HAN in leaf development was largely neglected. Here it was found that leaves of the *han-2* mutant in the Col background changed from serrated into smooth margins (Fig. 3G, H), Together with the finding that ectopic expression of *AtHAN* led to lobed leaves in *Arabidopsis* (Zhao *et al.*, 2004), a function for *AtHAN* in leaf shape development is hypothesized.

In this study, it was found that ectopic expression of *CsHAN1* can rescue the smooth margin phenotype in the *han-2* mutant and resulted in lobed leaves in WT *Arabidopsis* (Fig. 3). More importantly, both *CsHAN1-OE* and *CsHAN1-RNAi* lines produced highly lobed leaves in cucumber (Fig. 6A–F), especially in the leaves at the first 10 nodes (Fig. 6G). The *CUC* boundary genes have been shown to play a role in leaf development (Aida *et al.*, 1999; Nikovics *et al.*, 2006; Hasson *et al.*, 2011). In tomato, both reduction and overexpression of the *CUC* homologous gene *GOBLET* (*GOB*) led to a change from complex leaves in the WT into simpler leaves with no sharp leaf margin (Blein *et al.*, 2008; Berger *et al.*, 2009). These data suggest that the elaborate expression of the boundary genes *HAN* and *CUC* is essential for leaf shape development, with increased or reduced expression resulting in a change in leaf margins. However, molecular and genetic studies are required to establish whether HAN and CUC may be part of the same pathway or act independently in leaf development.

Further, the present data showed that despite the *CsHAN1-OE* lines and *CsHAN1-RNAi* lines displaying similar leaf phenotype, the underlying gene expression was different (Fig. 6). As a co-suppression event may be involved in the *CsHAN1-OE* lines and negative autoregulation of *AtHAN1* has been well documented (Zhang *et al.*, 2013), the final phenotypes of transgenic plants might derive from different levels of HAN proteins. The expression of *CsJAG*, *CsBP*, *CsKNAT2*, and *CsKNAT6* was down-regulated in the *CsHAN1*-*OE* lines (Fig. 6). Interestingly, *JAG*, *BP*, *KNAT2*, and *KNAT4* were also shown to be down-regulated upon *AtHAN* induction in *Arabidopsis* (Zhang *et al.*, 2013), suggesting that a similar regulatory mechanism may be involved between *HAN*, *JAG*, *BP*, and *KNOX* genes in cucumber and *Arabidopsis*. *CsBP* was found to be down-regulated in the meristem but up-regulated in the leaves of *CsHAN1-RNAi* cucumber plants, implying that different regulatory networks exist in different tissues and/or developmental stages.

### Partially conserved function of *CsHAN1* in flower development

The most obvious phenotype in the *han* mutant was the reduced floral organs (especially petals and stamens) and fused sepals in *Arabidopsis* (Zhao *et al.*, 2004). Despite the fact that ectopic expression of *CsHAN1* can partially rescue the floral organ and silique length phenotype of the *han-2* mutant (Fig. 3), no obvious flower organ defects were observed in either *CsHAN1-OE* or *CsHAN1-RNAi* lines in cucumber (data not shown), suggesting that *CsHAN1* may possess both conserved and divergent functions in flower development. Morphologically, the flowers in *Arabidopsis* and cucumber are quite different. *Arabidopsis* has bisexual flowers with separate sepals, petals, and stamens, and fused carpels in the innermost whorl (Supplementary Fig. S4A, B at *JXB* online). In cucumber, flowers are unisexual (male and female flower) with a tubular structure consisting of fused sepals, petals, stamens, or pistils at the base of a flower (Supplementary Fig. S4C–F). In *Arabidopsis*, *AtHAN* is transcribed at the boundaries between the meristem and its newly initiated organ primordia and at the boundaries between different floral whorls (Zhao *et al.*, 2004). Such boundary expression, especially in the boundaries between floral meristem and sepal primordia, or between sepal and petal primordia, was not observed for *CsHAN1* in cucumber (Fig. 2), suggesting that the boundary expression of *HAN* may be essential for floral organ separation and therefore affects organ numbers. Considering that there are two *HAN* homologues in cucumber (Fig. 1), lack of flower phenotype in the *CsHAN1* transgenic lines may due to the redundant role of *CsHAN2* in cucumber, or the function of the remaining *CsHAN1* in the knock-down lines (*CsHAN1-OE*). Future studies using the knock-out lines of both *CsHAN1* and *CsHAN2* through the CRISPR/Cas9 system (H. Zhang *et al.*, 2014) would be a promising way to dissect the specific function of *CsHAN* genes during the unisexual flower development in cucumber.

## Supplementary data

Supplementary data are available at *JXB* online.

Figure S1. *CsHAN1* overexpression in WT *Arabidopsis*.

Figure S2. PCR identification and qRT-PCR analyses of transgenic cucumber.

Figure S3. Leaf phenotype in the *CsHAN1* transgenic cucumber.

Figure S4. Flower morphology in *Arabidopsis* and cucumber.

Table S1. Primers used in this study.

Supplementary Data

## Abbreviations:

*AGO1*: *ARGONAUTE1*
*AS1/2*: *ASYMMETRIC LEAVES1/2*
*BP*: *KNAT1/BREVIPEDICELLUS*
CaMV: *Cauliflower mosaic virus*
CDS: coding sequence
*CLV1/2/3*: *CLAVATA1/2/3*
*CUC1/2/3*: *CUP-SHAPED COTYLEDON1/2/3*
FM: flower meristem
*HAN*: *HANABA TARANU*
IM: inflorescence meristem
*JAG*: *JAGGGED*
*KNAT1/2/6*: *KNOTTED1-LIKE HOMEOBOX GENE1/2 /6*
*KNOX*: *KNOTTED1-LIKE HOMEOBOX*
NJ: Neighbor–Joining
*NL1*: *NECK LEAF1*
OE: overexpression
*PNH*: *AGO10/PINHEAD*
SAM: shoot apical meristem
*STM*: *SHOOT MERISTEMLESS*
*TSH1*: *TASSEL SHEATH1*
WT: wild type
*WUS*: *WUSCHEL*
