# Supplementary material for: HANABA TARANU regulates the shoot apical meristem and leaf development in cucumber (Cucumis sativus L.)
Source: J Exp Bot. 2015 Aug 28;66(22):7075–87. doi: 10.1093/jxb/erv409 (PMC4765787; doi:10.1093/jxb/erv409)
Supplement: Supplementary Data [file supp_66_22_7075__index.html]

 HANABA TARANU regulates the shoot apical meristem and leaf development in cucumber (Cucumis sativus L.) — HANABA TARANU regulates the shoot apical meristem and leaf development in cucumber (Cucumis sativus L.) — Supplementary Data 

# *HANABA TARANU* regulates the shoot apical meristem and leaf development in cucumber (*Cucumis sativus* L.)

## Supplementary Data

Data files

- Supplementary Data - Supplementary Data
