# Supplementary material for: HANABA TARANU regulates the shoot apical meristem and leaf development in cucumber (Cucumis sativus L.)
Source: J Exp Bot. 2015 Aug 28;66(22):7075–87. doi: 10.1093/jxb/erv409 (PMC4765787; doi:10.1093/jxb/erv409)
Supplement: Supplementary Data [file supp_erv409_jexbot147306_file001.pdf]

Supplementary information for following article

HANABA TARANU regulates the shoot apical meristem and leaf development in cucumber (*Cucumis sativus L.*)

Lian Ding[1], Shuangshuang Yan[1], Li Jiang[1], Meiling Liu[1], Juan Zhang[1], Jianyu Zhao[1], Wensheng Zhao[1], Ying-yan Han [2], Qian Wang [1], and Xiaolan Zhang[1,#]

Figure S1

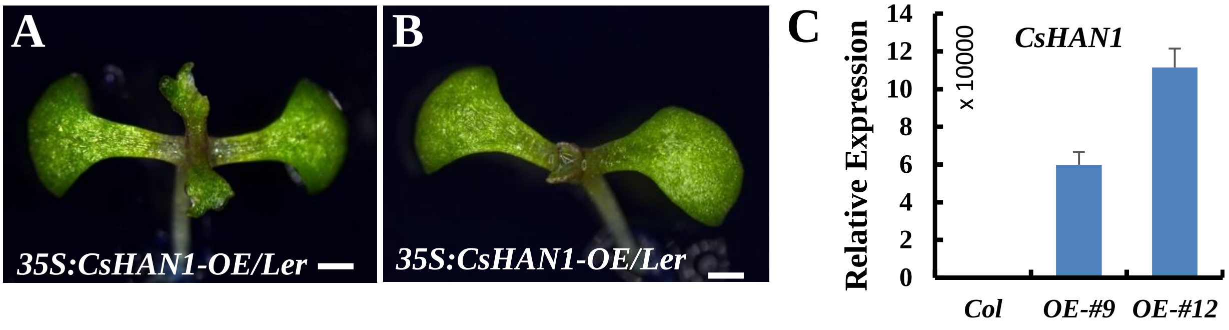

Supplemental Figure S1. *CsHAN1* overexpression in WT Arabidopsis. (A-B) The lethal phenotype of the two *CsHAN1* overexpression plants in *Ler* background. (C) qRT-PCR analyses of the *CsHAN1* expression in *Col* background.

Figure S2

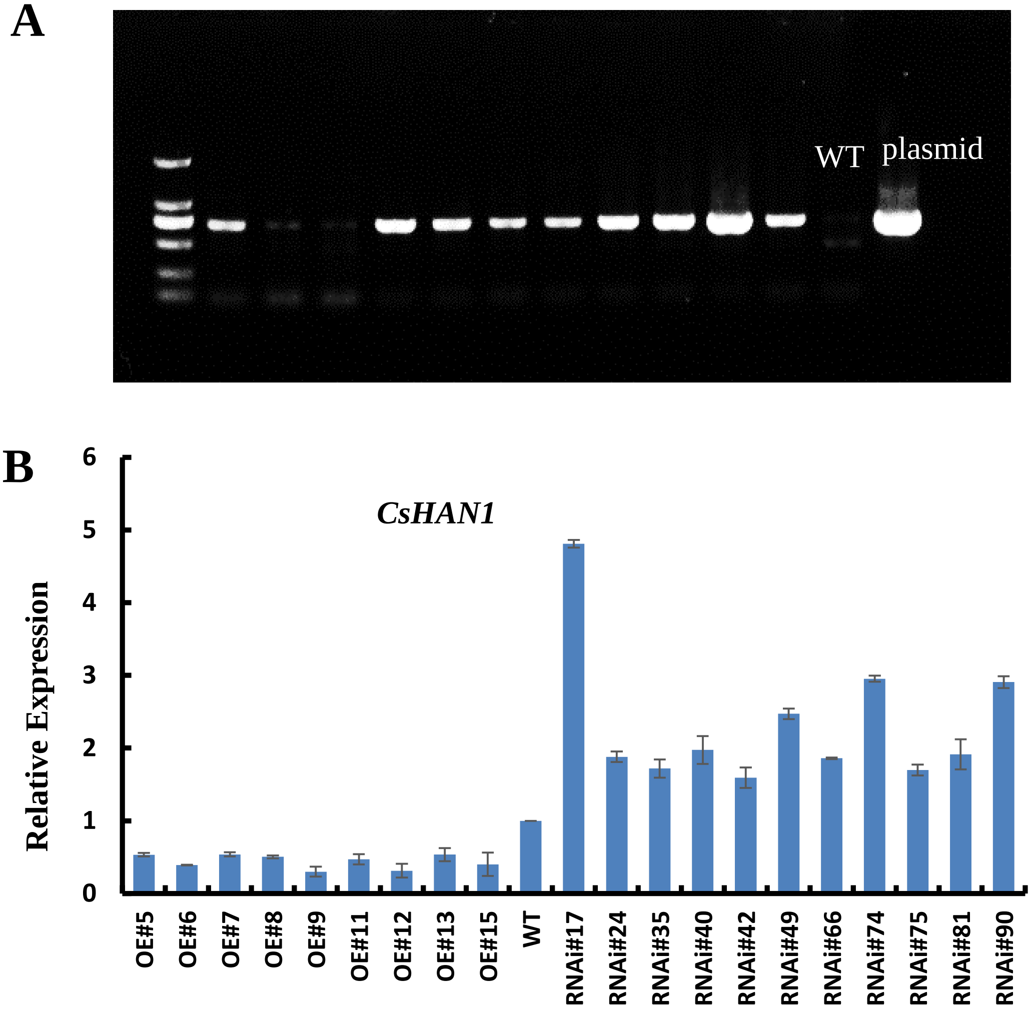

Supplemental Figure S2. PCR identification and qRT-PCR analyses of transgenic cucumber. (A) Representative gel picture of PCR identification of positive *35S:CsHAN1-RNAi* transgenic plants. (B) qRT-PCR analyses of the nine CsHAN1-OE and eleven CsHAN1-RNAi transgenic plants.

Figure S3

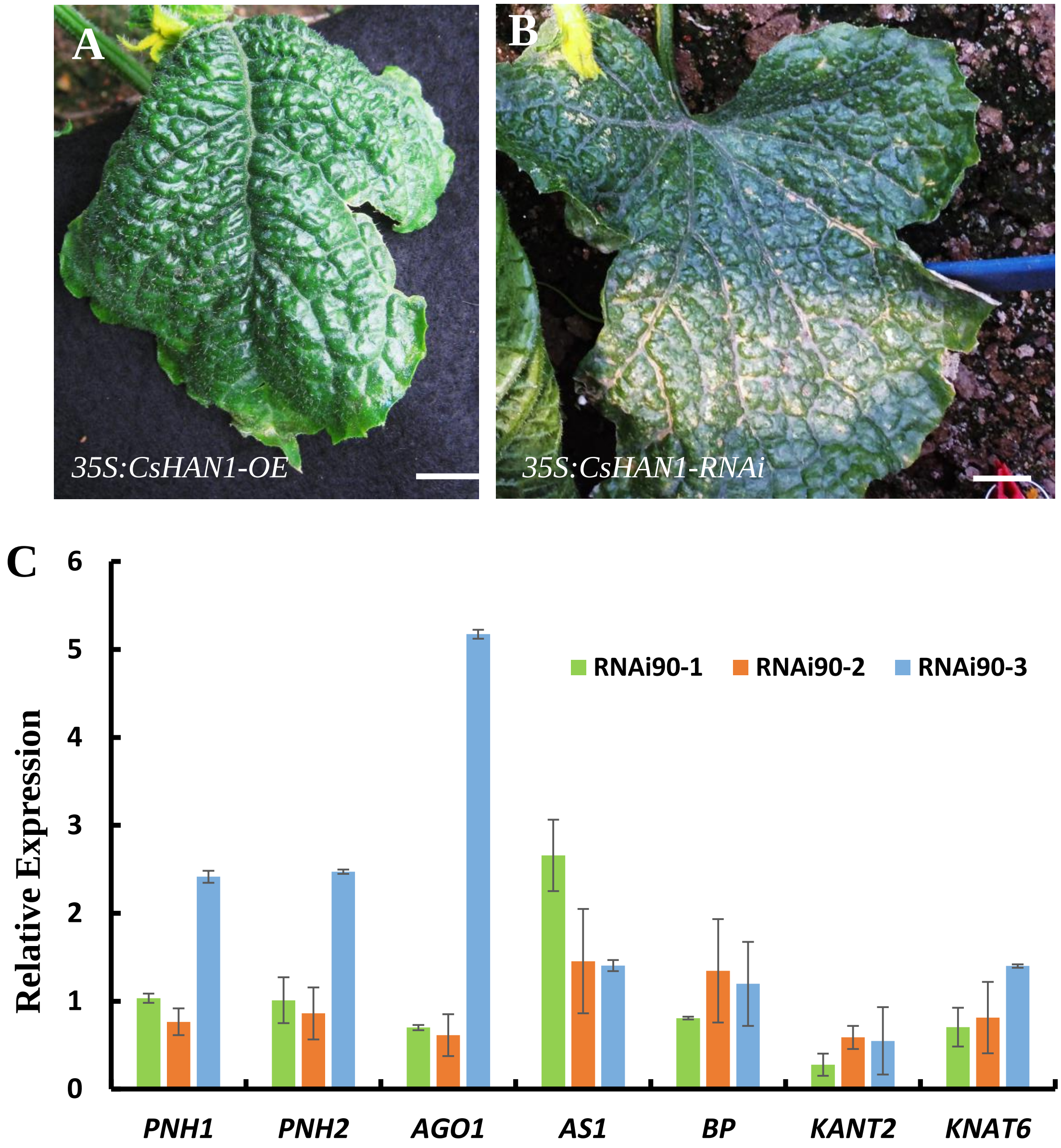

Supplemental Figure S3. Leaf phenotype in the *CsHAN1* transgenic cucumber. (A-B) The lobed leaves in the transgenic CsHAN1-OE line (A) and CsHAN1-RNAi line (B) showed in Figure 4B. (C) qRT-PCR analyses of leaf developmental genes in CsHAN1-RNAi line. The relative expression was calculated as the ratio of expression in lobed/normal leaves in the same plant.

Figure S4

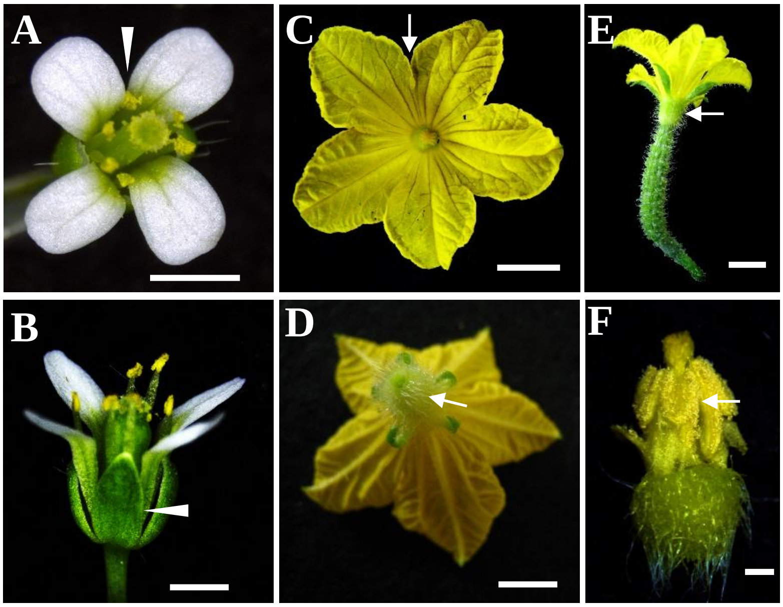

Supplemental Figure S4. Flower morphology in *Arabidopsis* and cucumber. (A-B) The top (A) and lateral (B) view of an *Arabidopsis* flower shows the separated floral organs. (C-D) The top (C) and dorsal view (D) of a cucumber male flower. (E) The side view of a cucumber female flower and (F) a male flower with the sepals and petals removed. Arrowheads show the separated floral organs in *Arabidopsis*, while the arrows indicate the fused floral organs in cucumber. Bars in (A-B) represent 1mm, in (C-F) represent 1cm.

Supplemental Table S1.   Primer information used in this study

**Primers for cloning**	
HAN 1S	5'-ATGATGCACCACTACGGCGG -3'
HAN 1A	5'-TCATCTTGTGAAATCATGGACGAGA -3'
HAN 2S	5'-ATGATGCACCACTACGGCG -3'
HAN 2A	5'-TCATCTTGTGAAATCATGGACGA -3'
**Primers for qRT-PCR**	
qHAN1-S	5'-GAACAGCTCGGTGGCAGAAT -3'
qHAN1-A	5'-GTCCAACGTCCTCGTTTCCT -3'
qHAN2-S	5'-GCTACAGCATCATTCTCATAG -3'
qHAN2-A	5'-CAACTTGTGAAATCATAATAAAG -3'
UBI-ep-F	5'-CACCAAGCCCAAGAAGATC-3'
UBI-ep-R	5'-TAAACCTAATCACCACCAGC-3'
qCsPNH1-S	5-TAAAGCCAACCACTTCCTAGC-3'
qCsPNH1-A	5'-TTACTTCCTCCATCATAAACCG-3'
qCsPNH2-S	5'-TCACCAAGGCAGGGGTAGA-3'
qCsPNH2-A	5'-GATGGATGGGAGGAAGAACTC-3'
qCsAGO1-S	5'-CCAGAAGGGCTACGACTGAAA-3'
qCsAGO1-A	5'-GGGTCAAATTGAACCAAGAAA-3'
qCsJAG-S	5'-TGATGAAGCAAAGGACAAGAAGA-3'
qCsJAG-A	5'-CGGTGGGTACACGACAAGAGA-3'
qCsAS2-S	5'-CTTCGTGACCCTGTCTACGG-3'
qCsAS2-A	5'-CGTGGTTACCAATCCCTAAGTT-3'
qCsBP-S	5'-AATGATGTAAATGAAAACTCAAATG-3'
qCsBP-A	5'-GTGAAGTAGTAGCTTCAGTCTTGAC-3'
qCsKNAT2-S	5'-CAAAACTATCTGTTACTACTGGTCTAG-3'
qCsKNAT2-A	5'-TCCAGTTTCTCCATTGTCATAAAA-3'
qCsKNAT6-S	5'-CCCGACAATCTTATCCTTCCC-3'
qCsKNAT6-A	5'-TGATGAGATCCGAACATAGGAATAG-3'
qJAG-S	5'- ATCTCTCCAAGTCCTAACCTCCC -3'
qJAG-A	5'- AGCGTCACCATCACGACCTTG -3'
qKNAT2-S	5'- TCGGAGAAAGCGACGTTGAT-3'
aKNAT2-A	5'- TCGAATACGCGGAAGCAACTC-3'
qSE-S	5'-CGCTTTATGGGTCGCTATCAG-3'
qSE-A	5'-AAAGGCACGTTTTTGTGTTGTGA-3'
qCBP20-S	5'-GCTAAGAGCATCTACAACGGTT-3'
qCBP20-A	5'-CAGGACGAAACAAAAGCCACA-3'
qAS2-S	5'-GTCATCTCTCTCCTCCAACATCA-3'
qAS2-A	5'-TGGCTGTTCCATCTGCTGCTC-3'
qSE-S	5'-CGCTTTATGGGTCGCTATCAG-3'
qSE-A	5'-AAAGGCACGTTTTTGTGTTGTGA-3'
qBP-F2	5'-AATAGTAGCAATTATGGTCCTGG-3'

qBP-R	5'-CTTGAGTATTGTGGATGGCTCT-3'
qCUC3-F	5'-GCTCAACGGTATCATCTTTCC-3'
qCUC3-R	5'-AACCCAACAGACCATAACTCG-3'
qCsWUS-S	*ATTCCACTTCTCCTATTACTACCTC*
qCsWUS-A	*GAACACTCTCTAAAACTGTTCTCAA*
qCsSTM-S	*GGTAATAATGGTGGTGGGTGT*
qCsSTM-A	*AGGGAGATGAGAGGAGGAAAT*
ACTIN2-F	5'-CCTTCGTCTTGATCTTGCGG-3'
ACTIN2-R	5'-AGCGATGGCTGGAACAGAAC -3'
**Primers for vector construction**	
HAN2 OE-S	5'-GCTCTAGAGCATGATGCAACGTTGTGGTAG-3'
HAN2 OE-A	5'-TCCCCCGGGGGATCAACTTGTGAAATCATAATAAAG-3'
HAN1 OE-S	5'-GCTCTAGAGCATGATGCACCACTACGGCG-3'
HAN1 OE-A	5'-TCCCCCGGGGGATCATCTTGTGAAATCATGGACGA-3'
HAN1-RNAi-A1S	5'-AAGGGATCCCTTTGAACAGCTCGGTGGCAGAA-3'
HAN1-RNAi -A1A	5'-AAGGGTACCCTTAGTGGGCCGGTGATCGGTA-3'
HAN1-RNAi -S1S	5'-TCGAGCTCGATGAACAGCTCGGTGGCAGAA-3'
HAN1-RNAi -S1A	5'-GGACTAGTCCAGTGGGCCGGTGATCGGTA-3'
**Primers for *in situ* probes**	
HAN1-sp6	5'-GATTTAGGTGACACTATAGaatGCTGGGATTCGATTCAAAAAGGAAGA-3'
HAN1-T7	5'-tgTAATACGACTCACTATAGGGTCATCTTGTGAAATCATGGACGAGA-3'
HAN2-sp6	5'-GATTTAGGTGACACTATAGaatGCTGCTACAGCATCATTCTCATAG-3'
HAN2-T7	5'-tgTAATACGACTCACTATAGGGCAACTTGTGAAATCATAATAAAG-3'
CsBP-SP6	5'-GATTTAGGTGACACTATAGaatGCTTGGAGGAATATAATAATGATGTAAA-3'
CsBP-T7-1	5'-tgTAATACGACTCACTATAGGGTCTGACATTCCATGTAGGCTT-3'
Cs STM-SP6	5'-GATTTAGGTGACACTATAGaatGCTTCTTGTTGTGAAGTAAAGGCTA-3'
Cs STM-T7	5'-tgTAATACGACTCACTATAGGGGAGGGAGATGAGAGGAGGA-3'
Cs WUS-SP6	5'-GATTTAGGTGACACTATAGaatGCTTTCTCCTATTACTACCTCCAATACT-3'
Cs WUS-T7	5'-tgTAATACGACTCACTATAGGGTGCAGAAACCACCGAGAT-3'
